# FedHealthFog: A federated learning-enabled approach towards healthcare analytics over fog computing platform

**DOI:** 10.1016/j.heliyon.2024.e26416

**Published:** 2024-02-16

**Authors:** Subhranshu Sekhar Tripathy, Sujit Bebortta, Chiranji Lal Chowdhary, Tanmay Mukherjee, SeongKi Kim, Jana Shafi, Muhammad Fazal Ijaz

**Affiliations:** a School of Computer Engineering, KIIT Deemed to be University, Bhubaneswar, 751024, India; b Department of Computer Science, Ravenshaw University, Cuttack, 753003, India; c School of Computer Science Engineering and Information Systems, Vellore Institute of Technology, Vellore, India; d Department of Computer Science and Engineering, Siksha ‘O' Anusandhan (Deemed to be) University, Bhubaneswar, 751030, India; e Department of Computer Engineering, Chosun University, Gwangju 61452, South Korea; f Department of Computer Engineering and Information, College of Engineering in Wadi Alddawasir, Prince Sattam Bin Abdulaziz University, Wadi Alddawasir, 11991, Saudi Arabia; g School of IT and Engineering, Melbourne Institute of Technology, Melbourne, 3000, Australia

**Keywords:** Federated learning, Healthcare, Fog computing, Performance evaluation, Latency, Energy efficiency

## Abstract

The emergence of federated learning (FL) technique in fog-enabled healthcare system has leveraged enhanced privacy towards safeguarding sensitive patient information over heterogeneous computing platforms. In this paper, we introduce the FedHealthFog framework, which was meticulously developed to overcome the difficulties of distributed learning in resource-constrained IoT-enabled healthcare systems, particularly those sensitive to delays and energy efficiency. Conventional federated learning approaches face challenges stemming from substantial compute requirements and significant communication costs. This is primarily due to their reliance on a singular server for the aggregation of global data, which results in inefficient training models. We present a transformational approach to address these problems by elevating strategically placed fog nodes to the position of local aggregators within the federated learning architecture. A sophisticated greedy heuristic technique is used to optimize the choice of a fog node as the global aggregator in each communication cycle between edge devices and the cloud. The FedHealthFog system notably accounts for drop in communication latency of 87.01%, 26.90%, and 71.74%, and energy consumption of 57.98%, 34.36%, and 35.37% respectively, for three benchmark algorithms analyzed in this study. The effectiveness of FedHealthFog is strongly supported by outcomes of our experiments compared to cutting-edge alternatives while simultaneously reducing number of global aggregation cycles. These findings highlight FedHealthFog's potential to transform federated learning in resource-constrained IoT environments for delay-sensitive applications.

## 1 Introduction

The rapid advancements in technology witnessed in the past twenty years have significantly expedited the expansion of data, resulting in issues associated with privacy of sentive data when stored in centralized systems [1]. This phenomenon is evident across all sectors, particularly in the healthcare industry, when institutions continue to retain patients' confidential data within centralized storage repositories. A more recent analysis of cyber-attack statistics found that 36 billion records were exposed due to data breaches in just the first half of 2020 [2]. Users' concerns about the privacy of their personally identifiable information, financial information, medical records, trade secrets, and other data types motivate the development of more advanced forms of artificial intelligence based on data privacy [3].

Because of the interconnected sensing, computing, and communicating capabilities of Internet of Things (IoT) gadgets, healthcare systems stand to benefit significantly from their implementation. Therefore, there has been a lot of interest in the concept of IoT-based wellness surveillance [4,5]. IoT gadgets, especially wearable ones like smartphones and smartwatches, can continuously monitor a user's movement, heart rate, and blood pressure and afterward send that information back to a healthcare facility for analysis and assessment. Smart in-home healthcare is becoming mainstream for smart healthcare [6] because of the simplicity and inexpensive nature of acquiring individual health information, allowing for ubiquitous and nonintrusive health surveillance.

Since services are hosted in geographically dispersed Cloud data centers [7]. Due to the time-critical nature of medical IoT real-time applications, cloud computing is impractical [8]. Fog or Edge computing is an extension of the Cloud that helps smart IoT devices utilize their resources more efficiently [9]. It paves the way for more granular control over the privacy and security of data generated by connected medical smart devices and computational resources [10]. Important Internet of Things applications that can't afford any lag in reaction time are also supported [11]. Getting the right call with the right tools in under a minute might mean the difference between life and death in certain medical circumstances [12].

Machine learning (ML) models trained on enormous user data are widely used in today's healthcare applications for insight generation, in-home health monitoring, and improving public health services and products. The ML technique known as Federated Learning (FL) includes keeping data samples locally and avoiding the transfer of information while training the considered algorithm across several decentralized servers or Edge devices [1]. Tens of thousands, if not millions, of remote devices, can provide data to a worldwide statistical model. With FL, devices like smartphones may build a centralized prediction model without relying on the Cloud to store training data. The final product is trained while powered down but connected to the internet through WiFi. With these gadgets' increasing processing power and concerns about data transmission security, local data storage and moving network processing to the network's periphery are gaining favor. Advantages of FL for IoT applications include improved data privacy, faster network communication, higher quality learning, and more [13].

To keep the quality of service (QoS) promises made to end users, demanded application services must be migrated from one processing node to another as end IoT devices move around. Because the user's proximity to some corresponding Fog node or Edge service may change when they move from one location to another, mobile users cannot reap the benefits [14]. When Internet of Things (IoT) devices often switch between access points, it can disrupt Fog computing infrastructure in real-time. Edge/Fog computing service provides intelligent services at the network's edge to satisfy the critical computing demands of IoT applications in real-time and meet the needs of latency and capacity on the network.

In order to facilitate connectivity, management, and execution over smart IoT devices, several different technologies are utilized by IoT applications. Recent years have seen a rise in popularity [15] for microservices, a subset of service-oriented architecture. Due to the narrow focus of each microservice, fewer computational resources and less communication overhead are needed to complete a given sub-task or service. Microservices' loosely linked modules offer benefits such as autonomous implementation, flexibility, and fault isolation [16], allowing them to expand up or down dynamically based on Fog nodes' resource availability and workload.

The purpose of this investigation is to lay the groundwork for FedHealthFog, a smart federated learning enabled healthcare system running over the fog computing platform for heart disease data in microservice-based IoT medical applications.

This study makes the following contributions to the field of study:

• The creation and implementation of the FedHealthFog framework have been the focus of our study with a fundamental goal of reducing the communication overheads that are necessary in the context of federated learning (FL).
• Creation of a distributed and decentralized FL environment that is tailored specifically for edge devices with limited resources.
• We have created and adopted a strategy based on a methodology known as greedy heuristics. At the end of each epoch, choosing the optimum global aggregator fog node is a crucial task that has been carefully developed and applied within our system. The primary objective of this specific method was to increase the dependability and reliability of the system.
• Our thorough examination of the FedHealthFog system includes both in-depth system analysis and simulation in realistic environments. With a particular focus on three crucial dimensions—test accuracy, communication latency, and the energy consumption profiles of the edge devices.
• Our evaluation also covers the area of communication latency, where we look into the lags in processing and transmitting data inside the framework.
• We examine the energy consumption trends displayed by the edge devices while functioning within the FedHealthFog ecosystem in addition to accuracy, loss value, and latency. This aspect of the evaluation offers a thorough grasp of the energy dynamics of the framework and its viability in scenarios with limited resources.

The remaining sections of this work are structured as follows. Sections 2, 3 Related Research and System model, respectively. Section 4 details the intended technique used in the experiments described in Section 5. The findings are discussed in Section 6, and the paper is concluded in Section 7.

## 2 Related work

Federated Learning uses the processing capacity of servers and data collected from dispersed devices, making it a good fit for Edge/Fog/Cloud computing use cases. To build a comprehensive global model, it is crucial to aggregate user models efficiently. The cornerstone strategy combines models from many clients to arrive at a fresh global average model. The improved model is redistributed to users for additional training. Federated Learning employs multiple aggregating methods to facilitate a worldwide model refresh.

Yuan et al. [17] offer a Fuzzy-gradient boosted decision tree (GBDT) algorithm for cardiovascular illness forecasting. The authors stated their suggested technique performed favorably in both binary and multiple classification predictions. Chakraborty and Kishor [18] demonstrated a cloud-fog-based IoMT system for cardiac disease prediction using machine learning classification techniques. To combine many medical images into one, a fusion network based on deep learning is proposed [19]. Since the suggested fusion architecture is unsupervised, custom fusion rules are unnecessary. Multiple healthcare applications are possible within the framework.

FedAvg is an effective, secure, private communication aggregation method proposed in FL over-edge devices. FedAvg removes clients who are sluggish to respond and assumes that everyone participates at the same rate [20]. The core of the FedMA aggregation method is a layer-by-layer learning process that finds and combines nodes with similar weights. Layers that have been trained separately communicate with the host computer [21]. FedProx solves the heterogeneity problem in federated networks by distributing the processing load evenly among all participating devices. This method promises a constant and exact convergence behavior by adding a proximal term to account for heterogeneity and the incorporation of partial information from lagging nodes [21]. The FedPer method separates the model into a specialized top layer and a generic bottom layer. The federated server aggregates the foundation layers utilizing transfer learning approaches [22] while the personalized levels are disconnected from the server. FedDist is an algorithm for federated learning that aggregates data using a similarity metric based on the Euclidean distance. The benefits of FedAvg and FedMA [23] are incorporated into this method. The total communication and processing expenses incurred by mobile devices can be reduced by decoupling the local update process from the global aggregation. Empirical tests further reveal that the proposed EdgeFed is more efficient than state-of-the-art techniques, cutting down on both the computational cost and the cost of connecting mobile devices, even when the available bandwidth varies. To accomplish this, mobile clients can send computational tasks to an Edge server.

Using FL for safeguarding confidentiality in IoMT contexts has been the focus of multiple works [25]. Decentralized, efficient, privacy-enhanced federated edge learning (DEEP-FEL) is a methodology suggested by Lian et al. [26] that uses federated edge learning in the healthcare industry. A federated deep learning system for biomedical monitoring was proposed by Can and Ersoy [27].

The researchers protected the privacy of individuals' cardiac activity recordings while using them to track stress levels. The framework's FL-based stress detection technique yields an accuracy of 81.75 percent. Adhikari et al. [28] presented deep transfer learning to identify contagious diseases in edge networks.

This technique takes the information learned by the trained model and creates a machine-learning model that is small enough to run on low-powered edge devices. The technology was able to predict diseases 99.8 percent of the time correctly. FedHome is a generative convolutional autoencoder (GCAE)-based architecture for in-home health surveillance presented by Wu et al. [29]. To protect the confidentiality of EHRs, Alzubi et al. [30] proposed a FL design that uses the cloud and blockchain technology. In Ref. [31], the authors suggest a customized FL system for clients with non-standard data distributions. Table .1 shows a comparison of different studies with respect to complexity, accuracy, cost, and speed.

**Table 1:** Comparison of different studies pertinent to our study.

Authored by	Complexity	Accuracy	Cost	Speed
Wang et al. [20],	Moderate	Moderate	Moderate	Moderate
Arivazhagan et al. [21],	Low	High	Low	High
Sannra et al. [22],	High	High	Moderate	Moderate
Randa M et al. [23],	High	Moderate	Moderate	High
Ye et al. [24],	High	High	High	High

## 3 3System model

**Fig. 1:**
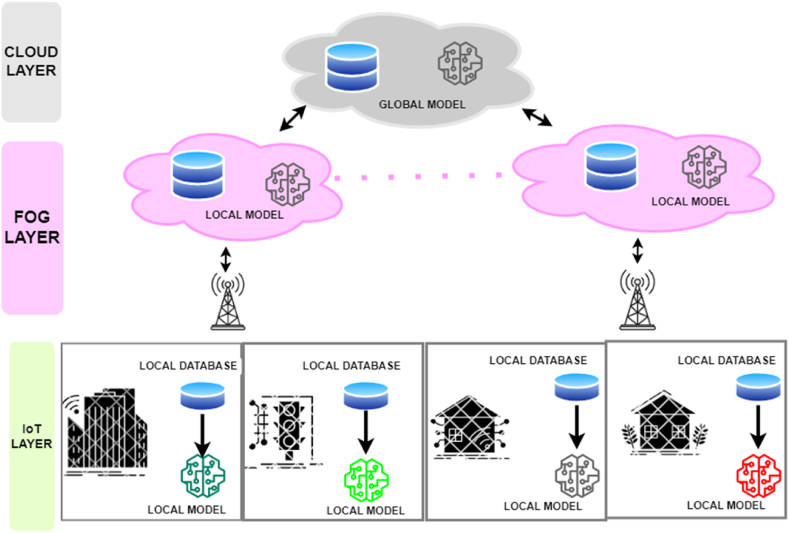
Architecture of the proposed FedHealthFog model.

In this section, we explain the intricate details of the FedHealthFog framework, an intriguing concept, and at the same time we present a meticulously developed greedy heuristic technique. This strategy has been carefully designed with the goal of discerningly choosing the best global aggregator fog device pertaining to each computation round. The FedHealthFog distributed federated learning system, introduced in this study and put into use, was created with resource-constrained IoT-enabled healthcare contexts in mind. In order to achieve partial aggregation of locally updated models from neighboring edge nodes, as shown in Fig. 1, our method inserts resource-rich fog nodes at critical geographic areas. Radio access network (RAN) technologies like WiFi are used to establish communication between devices. In the FedHealthFog framework, the cloud chooses the best fog node for global aggregation after a preset number of communication cycles and local aggregations involving edge and fog nodes. Fog nodes are an important addition to the device-to-cloud continuum because they mitigate potential bottlenecks and lessen the frequency of global aggregations during each cycle, which considerably improves the federated learning process. For edge devices utilizing IoT environments, this reduction translates into reduced communication latency and energy usage. FedHealthFog further reduces vulnerability by lowering dependency on a centralized entity during each epoch, improving system reliability. Additionally, it permits location-aware model training that is specifically designed for a subset of edge devices. Overall, FedHealthFog uses fog computing to handle resource limitations, reduce latency, save energy, and improve the efficiency and stability of the system in order to optimize federated learning for IoT-enabled healthcare systems.

**Fig. 2:**
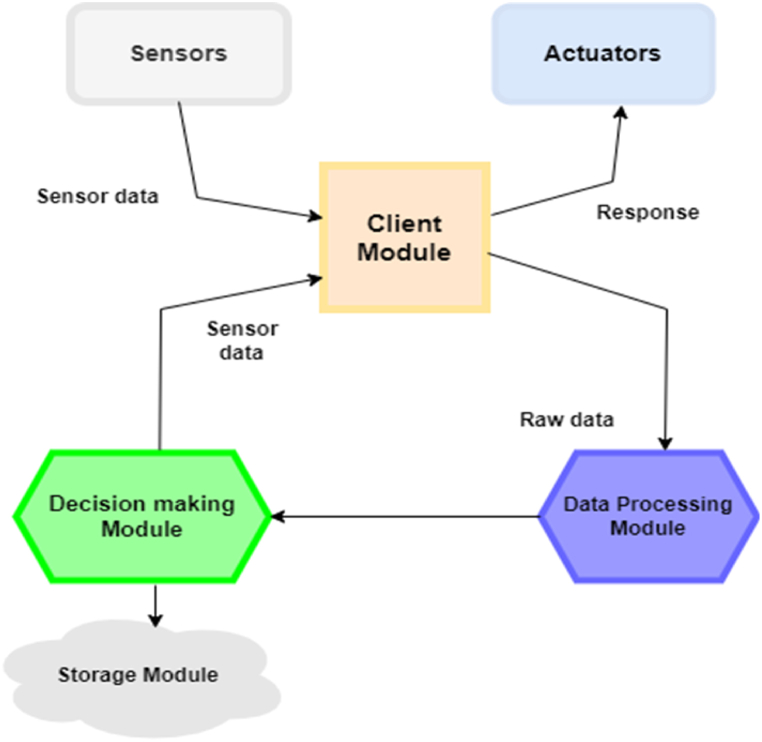
Data flow diagram of proposed model.

In Fig. 2, the FedHealthFog framework's workflow has been depicted through a detailed architecture made up of various modules. The sensor and actuator modules in charge of data collection and exterior interactions are at the core of the system. These modules supply data to the client module, which preprocesses the information before sending it to the data processing module for in-depth examination. Advanced algorithms are used by the data processing module to extract analytical information that is then passed on to the decision-making module for well-informed decisions. A closed-loop system that effectively manages sensor data, processing, decision-making, and storage is created when the results and pertinent healthcare data are finally saved in the storage module. This enables data-driven healthcare applications inside a fog-enabled ecosystem.

For the sake of our research, we take into account a network setup with κ edge devices acting as clients, along with F fog computing servers which serve as local computing servers in charge of both local and global aggregation. Apart from this, the network also considers a cloud server, which is deployed as the central coordinator node. Through a wireless channel interface, each edge node in a particular region establishes connection with the associated local fog node. The fog nodes also keep in touch with one another over a specific wireless channel. The fact that there are more edge nodes than fog nodes indicates a many-to-one mapping between the sets of edge nodes that are geographically close to one another κ and the sets of fog nodes F.

During each round of execution, the FedHealthFog framework's operational flow progresses through six distinct phases:

1.) *Selection:* The cloud node chooses and sets up a fraction C of clients to begin the round, where C∈(0,1).
2.) *Configuration:* The chosen clients are then set up to take part in the round M=⌈C∙κ⌉.
3.) *Local Update:* The model parameter ω must be updated locally by these clients.
4.) *Local Aggregation:* The system assumes that training data sets $\left\{D_{1},D_{2},\cdots ,D_{\kappa }\right\}$ are distributed unevenly and non-IID throughout the κ edge nodes. Every client κ processes a training data sample α made up of an input vector $X_{\alpha }$ and the intended output $Y_{\alpha }$ that corresponds to it. The loss function for each customer κ is then calculated and is denoted as $L_{\alpha }\left(X_{\alpha },Y_{\alpha },\omega \right)$ or just $L_{\alpha }\left(\omega \right)$.
5.) *Global Aggregation:* To calculate the total loss, the local loss functions from all clients are combined worldwide. Hence the local loss function corresponding to κ client processes can be given as Eq. (1),

(1) $$L_{\kappa }\left(\omega \right)=\eta_{\kappa }^{-1}\sum_{\alpha \in D_{1}}^{D_{\kappa }}L_{\alpha }\left(\omega \right)$$

where $\eta_{\kappa }=\left|D_{\kappa }\right|$ and represents the learning rate such that η≥0, and the local accuracy corresponding to each client process can be represented as 0≤θ≤1, here each of the κ client processes make an update to the parameter ω as given in Eq. (2) below:

(2) $$\omega_{\kappa }\left(\tau \right)=\omega_{\kappa }\left(\tau \right)-\eta \nabla L_{\kappa }\left(\omega_{\kappa }\left(\tau ,\beta_{\kappa }\right)\right)$$

where τ=0,1,⋯,N, which denoted the variation over time stamp τ, $\beta_{\kappa }$ represents the minibatch size of the model.

6.) *Reporting:* As part of the final phase, the results and updates are reported to present the final prediction outcomes after the model is trained over the considered dataset.

It is crucial to stress that the aforementioned steps collectively capture the fundamental workflow and functionality of the FedHealthFog framework within each iteration, making it easier to comprehend how it functions and how effective it is in situations with non-IID and unbalanced data distributions.

### 3.1 Local update phase

We approach the calculation of the number of local updates, denoted as $\mu_{\kappa }$, differently from the typical federated learning system described in Ref. [2]. Hence the number of local updated can be mathematically represented as,

(3) $$\mu_{\kappa }=\frac{E_{\kappa }\eta_{\kappa }}{\beta_{\kappa }}\times \frac{e_{\kappa }m_{\kappa }P_{\kappa }}{{Τ}_{\kappa }}$$

In our method, we consider a variety of device-specific variables as indicated in Eq. (3), like the residual energy $e_{\kappa }$, available amount of processing memory resource $m_{\kappa }$, the CPU capacity $P_{\kappa }$, and the quantity of tasks running simultaneously on a given device, indicated by ${Τ}_{\kappa }$. The number of local training instances $\eta_{\kappa }$, number of training runs $E_{\kappa }$, and size of minibatches $\beta_{\kappa }$ are three fundamental parameters of the federated learning process that are contrasted with these device conditions. After the necessary local updates are finished, the revised model parameters are collectively transmitted by all client nodes that are under the control of each fog node, denoted as F. The main goal of this transmission, which is aimed at the nearby fog node designated as F, is to make it easier to evaluate the local aggregation process.

We further compute the local aggregation parameter for the F nodes corresponding to τ th iterations as below:

(4) $$\omega_{F}\left(\tau \right)=\frac{\sum_{\kappa =1}^{Q_{F}}\eta_{\kappa }\omega_{\kappa }\left(\tau \right)}{\eta_{F}}$$

In the above Eq. (4), we refer to the varying number of clients which can be managed by a certain fog node, denoted by F, as $Q_{F}$, which is equal to the total of all κ clients: (from κ=1 to K) $=\sum_{\kappa =1}^{K}x_{F,\kappa }$. Here, the binary indicator variable $x_{F,\kappa }\in \left\{0,1\right\}$ denotes whether client κ is connected to fog node F. The total of the local dataset sizes across all clients under fog node F can be used to determine the number of local training examples within the scope of fog node F, abbreviated as $\eta_{F}≔\left(\kappa \to \left[1,Q_{F}\right]\right)$ $=\left|D_{\kappa }\right|$.

After the phases of client selection, setting-up their configuration, the local updates, and local aggregation are finished, the cloud server takes on the duty of choosing a global aggregator, designated as G, drawn from all of the fog nodes in set F. The evaluation of two crucial factors, workload and latency, served as the basis for choosing this worldwide aggregator. These variables play a crucial role in selecting the best fog node to carry out the ensuing global aggregation process.

We can hence obtain the global aggregator parameter by considering the average of local aggregation over each fog node F, and hence define it as below in Eq. (5):

(5) $$\tilde{\omega }\left(\tau \right)=\frac{\sum_{F}\omega_{F}\left(\tau \right)}{F}$$

The designated global aggregator node G then moves on to distribute the finished model to all fog nodes in the following step. The final step culminates in the storing of the model iteration on a cloud server, ready for use in the subsequent communication cycle. FedHealthFog's main goal is to incrementally improve the parameters of the global model, repeating this process until the global model reaches the required target accuracy, with the accuracy value limited to fall within the range of 0–1 in FedHealthFog.

**Figure:**
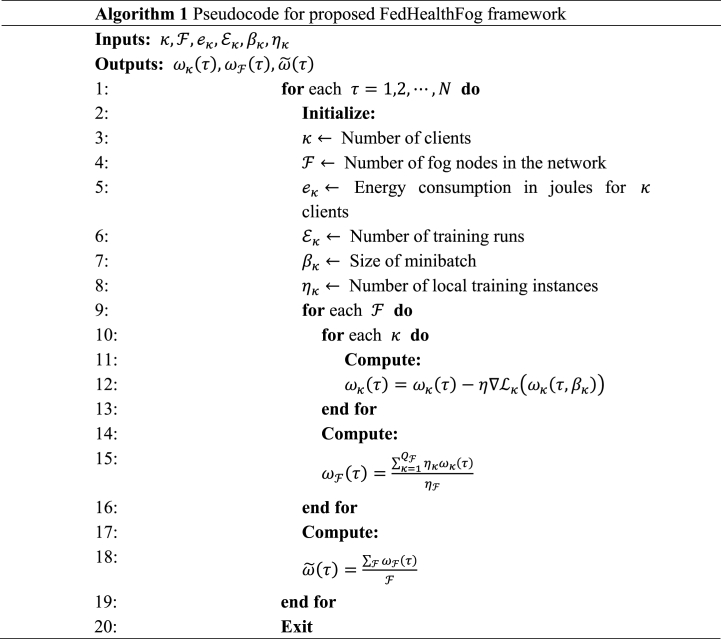

In Algorithm 1, the steps of the FedHealthFog framework's process are precisely outlined. It is crucial to remember that the framework includes $\mu_{\kappa }$ local updates as well as local aggregations $\tilde{\omega }\left(\tau \right)$ prior to executing one global aggregation step and hence provides desired prediction accuracy values of the considered dataset.

### 3.2 Global aggregation phase

The cloud server performs the crucial role of selecting a global aggregator node, designated as G, from the collection of accessible fog nodes in set F, during each operating cycle. The workload and communication latency are two crucial factors in this selection process' evaluation. These metrics act as important determinants in the thorough evaluation carried out to choose the best fog node for the function of global aggregation in the particular round.

#### 3.2.1 Estimation of workload

The expression for workload $\varphi_{i}\left(\tau \right)$ describes the quantity of data processing requests, quantified in bits, coming from the clients associated with each fog node indicated as i belonging to the set F during the τ th epoch. The following Eq. (6) is how this formulation is expressed:

(6) $$\varphi_{i}\left(\tau \right)=\sum_{\kappa =1}^{K}x_{i,\kappa }\times s,\forall i\in F$$

In the above Eq. (6), the value $x_{i,\kappa }$ considers a binary parameter that is illustrative of the connection between the κ th client and i th fog node in the network. The parameter s refers to the size of the locally updated model, and is considered to hold the same value for all clients in the system.

#### 3.2.2 Estimation of communication latency

A crucial factor that describes the time delay experienced during data transmission and exchange between fog nodes, edge devices, and cloud servers is communication latency in fog computing platforms. It is necessary to thoroughly assess the network structure, data transmission rates, propagation dimensions, and processing power of fog nodes in order to estimate communication delay in a fog computing system. To quantitatively forecast communication delay, advanced modeling approaches like queuing theory and network simulation are frequently used. This enables system designers to optimize resource allocation and assure timely data processing and decision-making in fog computing architectures.

The transmission and propagation delays that are present on each individual one-hop communication link between the i th and j th fog nodes can be explained as follows:

1.) *Transmission Delay:* The transmission delay $l_{i,j}^{trans}\left(\tau \right)$ is expressed as the ratio between the consistent model size maintained across all fog nodes s and the data transmission rate denoted as r. All fog nodes are believed to have the same rate. It can mathematically be denoted as Eq. (7) below:

(7) $$l_{i,j}^{trans}\left(\tau \right)=\left(s/r\right)$$

2.) *Propagation Delay:* The spatial spacing between the i th and j th fog nodes, represented as $\delta_{i,j}$, and the speed of light, represented as c, determine the propagation delay, indicated in Eq. (8) as,

(8) $$l_{i,j}^{prop}\left(\tau \right)=\left[\left(\delta_{i,j}\times s\right)/c\right],\forall i,j\in F,i\neq j\text{.}$$

The processing delay experienced at fog node represented as, $l_{i}^{F}$, where i belongs to the set F and is calculated in Eq. (9) as follows:

(9) $$l_{i}^{F}\left(\tau \right)=\sum_{\kappa =1}^{K}x_{i,\kappa }\left(z/\rho_{i}^{F}\right)$$

where z stands for the amount of CPU cycles needed to process the uniform model size s and $\rho_{i}^{F}$ stands for the CPU frequency unique to the i th fog node.

Following [[32], [33], [34]], we assume that the task arrivals to a certain fog node i within set F follow a Poisson process, which is represented by the parameter $\lambda_{i}$, which is the sum of all client processes with the fog node in the federated framework and can be given as Eq. (10) below:

(10) $$\lambda_{i}=\sum_{\kappa =1}^{K}x_{i,\kappa }$$

Furthermore, we model every fog node in set F using an M/M/1 queuing model, and the resulting queuing delay, $l_{i}^{que}\left(\tau \right)$, is calculated as Eq. (11),

(11) $$l_{i}^{que}\left(\tau \right)=\left[\frac{1}{\left(\sigma_{i}-\lambda_{i}\right)}\right]$$

where $\sigma_{i}$ is the service rate that applies to the i th fog node. The temporal aspects of communication and processing delays within the FedHealthFog architecture are explained in detail by this thorough exposition in Eq. (12) as below:

(12) $$l_{i}\left(\tau \right)=l_{i}^{D}\left(\tau \right)+l_{i}^{F}\left(\tau \right)+l_{i}^{que}\left(\tau \right)$$

where $l_{i}\left(\tau \right)$ is the total delay incurred across the entire fog network, and $l_{i}^{D}\left(\tau \right)$ is the latency delay to build the model across the entire sample size of the local dataset D, which can be defined as $l_{i}^{D}\left(\tau \right)=\max\left(l_{i,j}^{trans}\left(\tau \right),l_{i,j}^{prop}\left(\tau \right)\right),\forall i,j\in F,i\neq j$.

A unique fog node, designated i also in the set F, must be chosen in order to identify the global aggregator node, indicated as G and belonging to the set F, for each iteration. This choice is subject to the constraint that the selected fog node i exhibits the minimal values for both workload $\varphi_{i}\left(\tau \right)$ and delay $l_{i}\left(\tau \right)$. As a result, the formal definition of the utility function for the ith fog node, designated as $U_{i}$, is expressed in Eq. (13) as follows:

(13) $$U_{i}=\alpha l_{i}\left(\tau \right)+\left(1-\alpha \right)\varphi_{i}\left(\tau \right),\forall i\in F$$

To regulate and adjust the relative importance of both workload and delay inside the utility function, a constant parameter labeled as α is used in Eq. (13). It establishes the weighting that is given to each of these criteria in particular.

## 4 Problem formulation

The maximum permissible latency within the fog network is one of the main restrictions in the issue formulation of the FedHealthFog architecture. It states that the latency encountered by fog nodes must not go over a specific limit, or $l^{\max\;}$. When it comes to healthcare applications, where rapid judgments are frequently required, this limitation is essential for assuring prompt data processing and response times. A workload limitation is also included in the FedHealthFog framework's problem formulation. This restriction establishes a maximum workload for each fog node, preventing overuse of the available computational resources. This is crucial in healthcare settings as its crucial to properly manage resource allocation in order to ensure the timely and accurate processing of patient data.

Our goal is to minimize the utility function $U_{i}$, which represents the utility of the i th fog node in set F, in order to choose the best fog node to act as the global aggregator node. Maintaining low latency and following workload restrictions are two essential limitations that must be followed in order to achieve this minimization. In order to formalize this optimization project, we develop the below problem mathematical formulation by exploiting integer linear programming (ILP) principles, which captures our main objective:

(14) $$\arg\min_{i\in F}U_{i}=\alpha l_{i}\left(\tau \right)+\left(1-\alpha \right)\varphi_{i}\left(\tau \right)$$

s.t.,

(14a) $$l_{i}\left(\tau \right)\leq l^{\max\;}\text{,}$$

(14b) $$\varphi_{i}\left(\tau \right)\leq \varphi^{\max},\forall i\in F$$

Two fundamental constraints are developed to control the choice of the global aggregator node inside the optimization framework shown in Eq. (14). These constraints are:

1.) **Constraint (14):** The greatest allowable delay that can occur at any fog node inside the fog network is capped by constraint (14a). It makes sure that the delay stays below a predetermined limit, marked as $l^{\max\;}$.
2.) **Constraint (14b):** This restriction ensures that the workload stays below a predetermined threshold value denoted as $\varphi^{\max}$ by setting an upper limit on the maximum permissible workload at each fog node as obtained from Eq. (14b).

In this research study, the optimization problem specified in Eq. (14), is solved using a focused strategy. To address and optimize the choice of the global aggregator node, we specifically use a greedy heuristic methodology, which reduces the computational complexity involved with this problem. Algorithm 2 presents the workflow of the proposed greedy heuristic approach for the FedHealthFog framework. Following [11], it has been observed that the conventionally used greedy algorithm relies on optimal local decision making and does not take into account the golabl context. Further, it also relies on a single criterion, which makes the choice of global aggregator more static. The proposed greedy heuristic algorithm focuses on enhancing the efficiency of the adopted federated learning approach by incurring a strategic selection of nodes that aggregates and are responsible for coordinating updates in the model from different local fog nodes F. The algorithm specifies the nodes to which more priority should be given on their computational capacities, communication capabilities and proximity toward other nodes. The following pseudocode describes the main stages of this greedy heuristic incorporated into the FedHealthFog framework.

**Figure:**
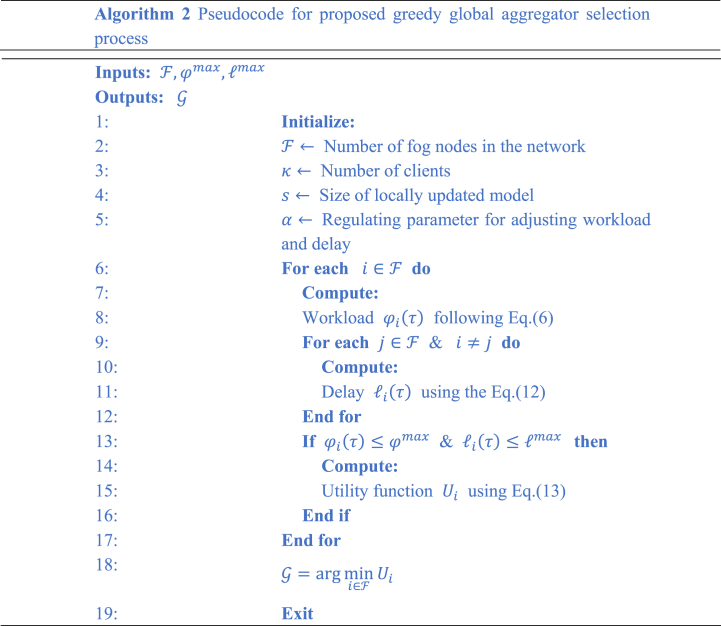

## 5 Performance evaluation strategies

We conduct a thorough examination of the proposed FedHealthFog framework in this section, concentrating on two crucial elements:

1.) *Delay Analysis:* We examine the delay properties of the FedHealthFog framework in this inquiry. We focus on the temporal aspects in particular, analyzing elements like communication lag, processing delays, and queue delays. We intend to acquire insights on the framework's time-related performance, which is crucial in applications where quick data processing and decision-making are essential.

According to earlier research [8], the length of FL processes, sometimes known as FL time, depends on the interaction of computing and communication elements in the network. The computational aspect concerns the time spent on local processing, which depends on elements like data size and the speed at which edge nodes can complete processing tasks. On the other hand, the ability to communicate depends on the network channel bandwidth and the distance between participating nodes.

To further analyze these elements, we begin by calculating the computation delay for the κ th fog node, abbreviated as $d_{\kappa }^{F}$. This computation delay is defined as, $d_{\kappa }^{F}=\left(\frac{z}{\rho_{\kappa }^{e}}\right)$, where z stands for the quantity of CPU cycles needed to process the data $D_{\kappa }$ and $\rho_{\kappa }^{e}$ stands for the frequency of the κ th fog node's CPU cycles. It is important to note that we assume that all fog nodes have the same model size s and data transmission rate r. As a result, over the entire system, the uplink transmission delay, $d_{\kappa }^{tran}$, is expressed as $d_{\kappa }^{tran}=\left(\frac{s}{r}\right)$.

In addition, the propagation delay for the uplink channel pertaining to the κ th edge node is estimated as, $d_{\kappa }^{prop}=\left[\frac{\left(d_{\kappa ,F}^{e}\times s\right)}{c}\right]$, where $d_{\kappa ,F}^{e}$ stands for the spatial distance between the κ th edge node and the associated fog node designated as F, and c stands for a constant that denotes the speed of light.

It is notable that downlink communication follows a similar calculating process for transmission and propagation delays. However, we view the downlink transmission time as insignificant in comparison to the uplink transmission time based on the findings of the study made in Ref. [8]. This is primarily explained by the high downlink bandwidth as well as the fog nodes’ transmission power operating with high performance configurations as opposed to edge nodes, which are resource-constrained.

Local aggregations are done before a single global aggregation in the context of the FedHealthFog framework, effectively assisting in the global model training over the course of N communication rounds. As a result, the total delay experienced at the κ th fog node during the i th global and j th local aggregation round is calculated as, $d_{i,j,\kappa }=d_{\kappa }^{prop}+d_{\kappa }^{tran}+d_{\kappa }^{F}$. Additionally, the collective edge-to-fog communication latency at this point is calculated as, $d_{i,j}^{\prime }=\max_{\kappa }\left(x_{i,j,\kappa }\times d_{i,j,\kappa }\right)$, where $x_{i,j,\kappa }$ signifies the edge node's selection status for the i th global aggregation round and j th local aggregation round.

Therefore, the mean delay corresponding to each communication round of the proposed framework can be defined as Eq. (15),

(15) $$d^{\prime \prime }=\frac{1}{N}\sum_{i=1}^{n}\sum_{j=1}^{m}d_{i,j}^{\prime }$$

2.) *Energy Consumption:* As part of our investigation, we focus on examining the patterns of energy consumption displayed by resource-constrained edge nodes working inside the FedHealthFog framework. Our scrutiny aims to provide a thorough understanding of the energy dynamics and efficiency implications associated with the FedHealthFog architecture. This aspect of the assessment is essential in contexts where energy efficiency and sustainable operation are prime concerns, such as in scenarios involving devices with limited power resources.

The FedHealthFog framework's energy consumption analysis consists of two independent parts and is mostly focused on the edge nodes' resource limitations.

a) **Energy consumed for computation**: The analysis of the total energy usage across all κ edge nodes during the i th global aggregation cycle and the j th local aggregation phase is based on the work done by Ref. [15]. Thus, the energy consumption for performing the computation tasks in the fog environment can be stated as Eq. (16) below:

(16) $$E_{i,j}^{comp}=\sum_{\kappa =1}^{K}\left[\frac{C_{\kappa }}{2}\times \left(z\times \rho_{\kappa }^{e}\times \eta_{\kappa }\times x_{i,j,\kappa }\right)\right]$$

where $C_{\kappa }$ represents the coefficient for effective capacitance, the number of CPU cycles required to process a data sample, denoted as $D_{\kappa }$, are shown as z, the number of CPU cycles specific to the κ th edge node is shown as $\rho_{\kappa }^{e}$, the number of data samples processed is shown as $\eta_{\kappa }$, and $x_{i,j,\kappa }$ is a binary variable that indicates whether edge node κ participates in the i th global aggregation and j th local aggregation round.

b) **Energy consumed for communication:** We utilize the energy model developed by Heinzelman et al. [35] in order to determine the overall energy consumption associated with communication. The energy used for transmission and amplification across the κ edge nodes during the i th global and j th local aggregation round is taken into account by this model [36,37]:

(17) $$E_{i,j}^{comm}=\sum_{\kappa =1}^{K}\left[x_{i,j,\kappa }\left(E_{i,j,\kappa }^{tran}\times E_{i,j,\kappa }^{amp}\times s\times d_{\kappa ,F}^{e}\right)\right]$$

In Eq. (17), $E_{i,j,\kappa }^{tran}$ denotes the energy for transmission and $E_{i,j,\kappa }^{amp}$ represents the energy for amplification over the κ nodes respectively.

Hence, the average energy consumption for the proposed FedHealthFog network can be determined by exploiting Eq. (16) and Eq. (17) as Eq. (18) below:

(18) $$E^{total}=\frac{1}{N}\sum_{i=1}^{n}\sum_{j=1}^{m}\left({E_{i,j}^{comP}+E}_{i,j}^{comm}\right)$$

## 6 Results and discussions

### 6.1 Experimental setup

We have extensively built a simulation environment to support a significant number of operational nodes in order to precisely recreate the fundamental characteristics of our experimental setup. The foundation for executing a variety of experiments, with deliberate adjustments in factors like client fractions, fog node designs, and the number of local aggregations, is this precisely constructed simulation environment within MATLAB R2020a. We have used a computer system with an Intel i5 processor running at 3.2 GHz and 8 GB of RAM to make these experiments possible. Complex computations and simulations may be carried out effectively thanks to this reliable computational infrastructure. We used a consistent strategy to randomly distribute the operational nodes throughout a spatial coverage area that is 5×5 km. sq. in the FedHealthFog environment. We have varied the number of clients between 5 and 20, while maintaining a constant overall population of edge nodes throughout all experimental scenarios.

We've included a comparison analysis of the proposed FedHealthFog framework with three existing benchmark algorithms: Hierarchical Fog-Cloud FL, Federated Averaging, and Fed-SGD as part of our thorough performance analysis. These benchmarks have been put through several performance evaluation tests carried out in the simulated environment, allowing us to make insightful conclusions and contrasts on many aspects of the functionality and effectiveness of the framework.

### 6.2 Performance evaluation and synthesis

**Fig. 3:**
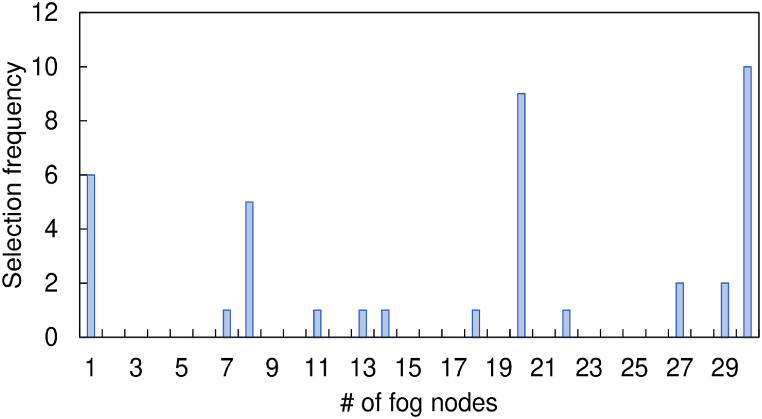
Number of fog nodes' selection frequency corresponding to the global aggregator.

The result shown in Fig. 3 sheds light on how frequently different fog nodes are chosen as global aggregator nodes throughout each operational round. This illustration offers concrete proof of the dynamic nature brought about by our suggested greedy heuristic approach to the selection procedure. In particular, it illustrates the framework's flexibility in selecting various fog nodes that have low workload and latency on a round-by-round basis. The worst-case scenario, in which none of the available fog nodes can handle the required high workload and latency requirements, must be taken into account. In these situations, the cloud server is in charge of carrying out the global aggregation process. This backup method guarantees the operation's continuation even if it deviates from the normal fog-based aggregation strategy, highlighting the framework's resilience under challenging circumstances.

**Fig. 4:**
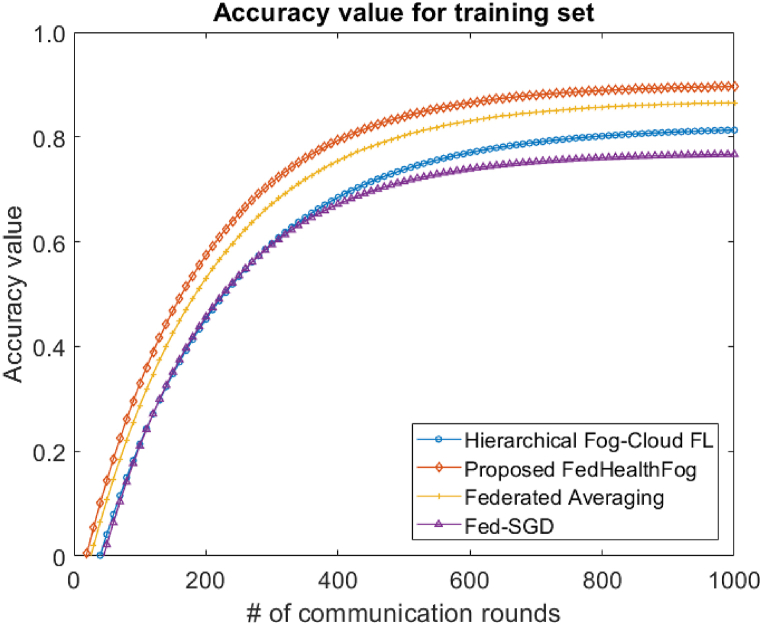
The comparison plot for different number of communication rounds with accuracy value for the training set.

**Fig. 5:**
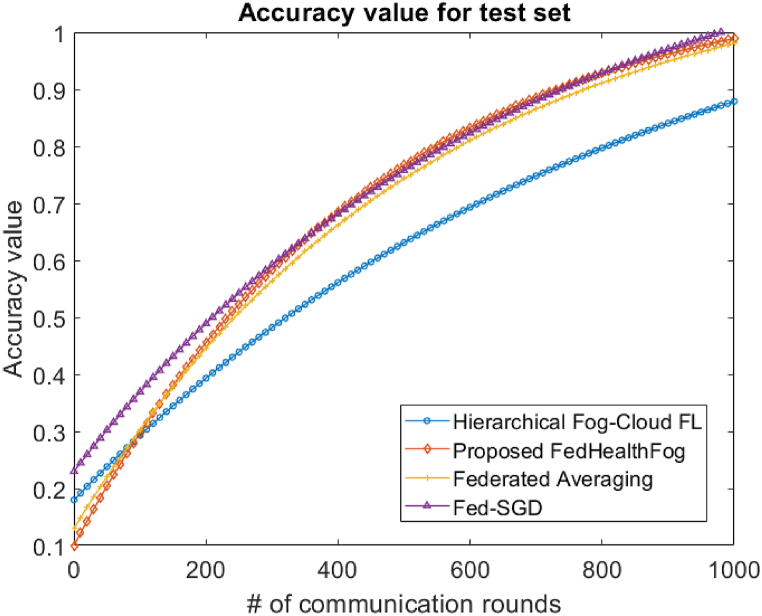
Comparison of different communication rounds with accuracy value for test set.

**Fig. 6:**
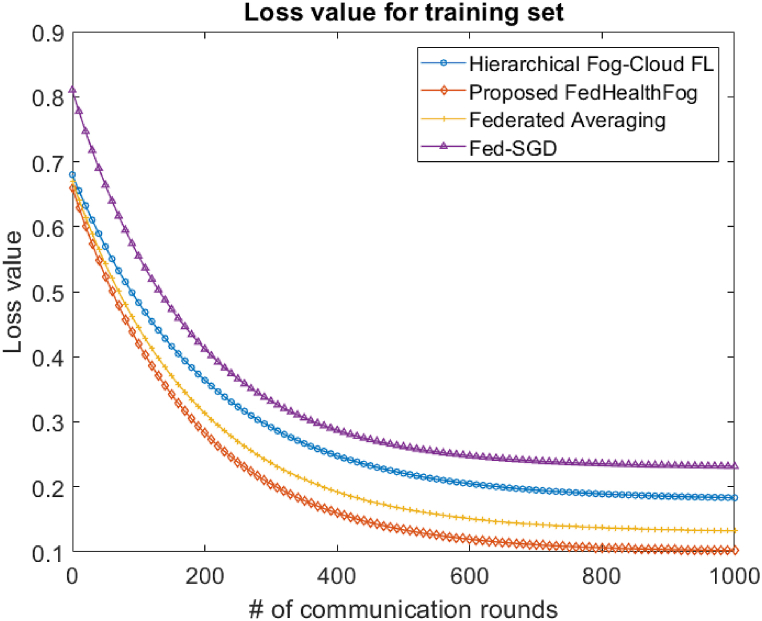
Comparison of communication rounds with loss value for training set.

**Fig. 7:**
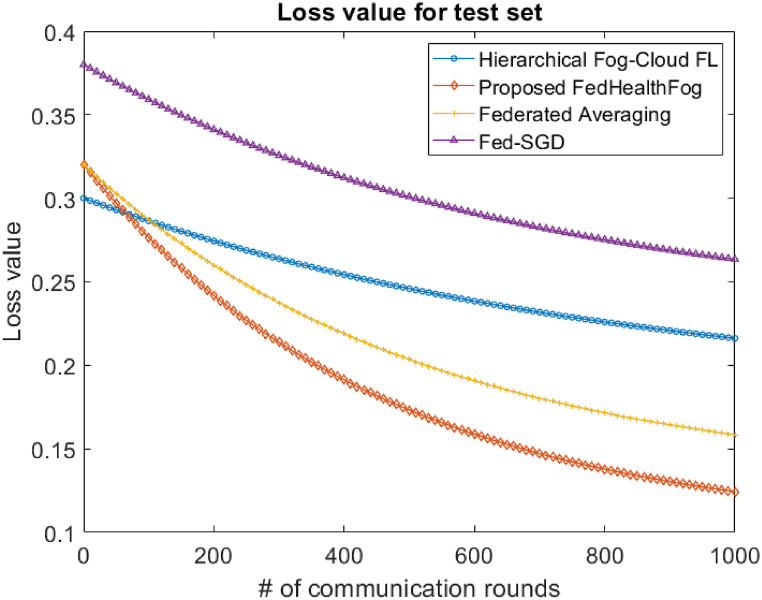
Comparison of different communication rounds with loss value for test set.

With the Hierarchical Fog-Cloud FL, Federated Averaging, Fed-SGD, and proposed FedHealthFog frameworks, as well as variable numbers of local aggregations, set at 10 and 20, we have conducted an extensive analysis to evaluate the impact of multiclient parallelism. The accuracy charts displayed in Fig. 4, Fig. 5 are the foundation for our assessment. These charts provide evidence that FedHealthFog framework routinely demonstrate worldwide test accuracy levels that are strikingly comparable to and noticeably better than those seen in the case of benchmark algorithms. This finding emphasizes how well FedHealthFog perform when compared to the traditional techniques. We have determined the necessary number of communication rounds in addition to evaluating accuracy in order to meet the desired accuracy criterion of 0.91. This computation has been done for all three frameworks with a range of client fractions, illuminating the effectiveness of these methods in achieving the targeted performance milestone over varying communication rounds up to 1000. Further, in Fig. 6, Fig. 7, the loss values for the training set and test set are obtained respectively. It was observed from the graphical representation that with the increase in the number of communication rounds, the loss value for the proposed FedHealthFog framework reduced considerably.

**Fig. 8:**
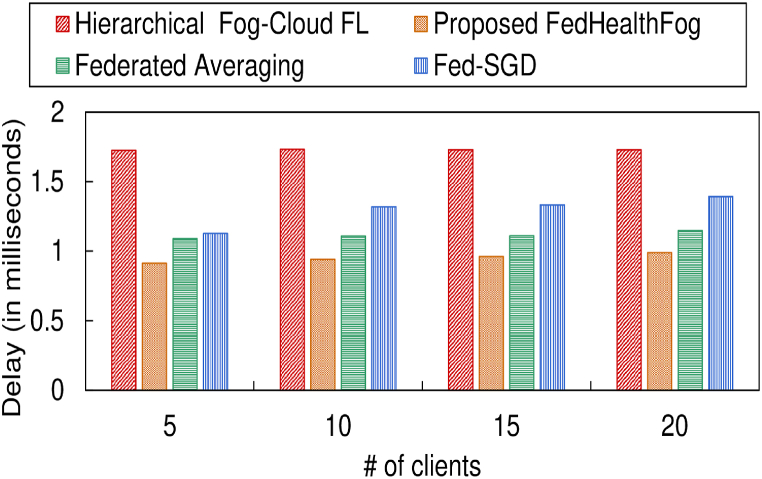
Comparison of different number of clients with delay for training set.

**Fig. 9:**
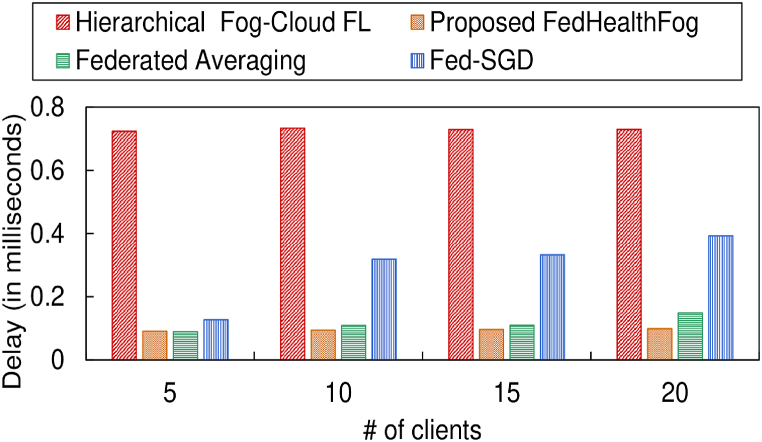
Comparison of different number of clients with delay for test set.

In Fig. 8, Fig. 9, the delay incurred by the 4 models corresponding to the training set and test set has been depicted respectively, over varying number of clients in the federated learning environment. Table 2 provides a summary of the experimental outcomes for delay incurred by the proposed FedHaelthFog model with other models. It can be observed that for the training set, the proposed FedHealthFog model incurred an average percentage decrease in delay of 44.97%, 14.64%, and 26.41% from the benchmark models i.e., Hierarchical Fog-Cloud FL, Federated Averaging, and Fed-SGD respectively. Further, the FedHealthFog model showed decrease in delay for the test set as 87.01%, 26.90%, and 71.74% respectively, for the Hierarchical Fog-Cloud FL, Federated Averaging, and Fed-SGD models. It was observed that the delay incurred by the proposed FedHealthFog framework was the minimal as compared to Hierarchical Fog-Cloud FL, Federated Averaging, and Fed-SGD framework.

**Table 2:** Summary of delay value in milliseconds for training set and test set over varying number of clients pertaining to different models.

Models used	No. of Clients (for Training Set)			
	5	10	15	20
Hierarchical Fog-Cloud FL	1.7236	1.7329	1.7286	1.7294
**Proposed FedHealthFog**	0.9125	0.9412	0.9621	**0.9893**
Federated Averaging	1.0892	1.1096	1.1103	1.1486
Fed-SGD	1.1269	1.3188	1.3325	1.3924
	**No. of Clients (for Test Set)**			
	**5**	**10**	**15**	**20**
Hierarchical Fog-Cloud FL	0.7021	0.7141	0.7308	0.7731
**Proposed FedHealthFog**	0.0912	0.0928	0.0940	**0.1013**
Federated Averaging	0.0933	0.1128	0.1342	0.1786
Fed-SGD	0.1614	0.3631	0.3943	0.4236

**Fig. 10:**
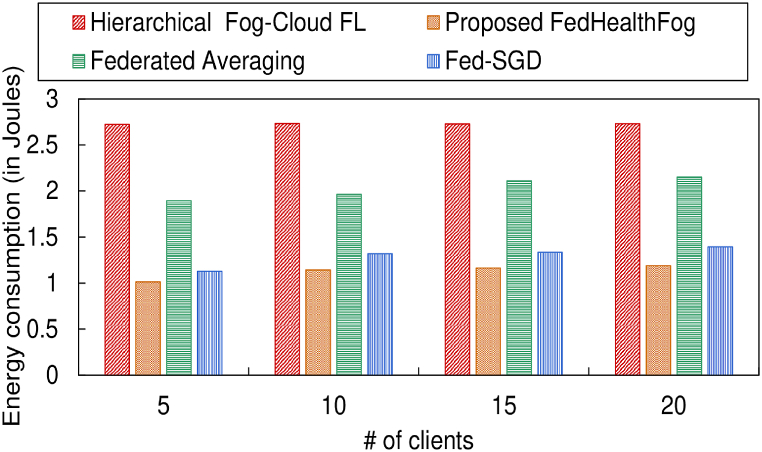
Comparison of different number of clients with energy consumption for train set.

**Fig. 11:**
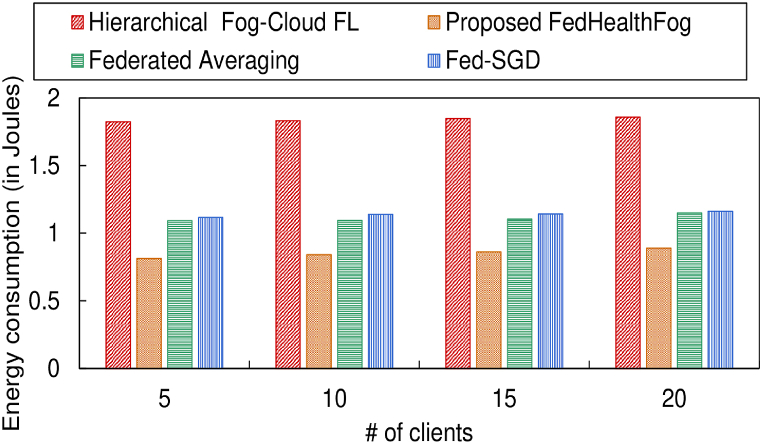
Comparison of different number of clients with energy consumption for test set.

Also in Fig. 10, Fig. 11, the energy consumption for the 4 models corresponding to the training set and test set has been provided respectively, over varying number of clients in the federated learning environment. Table 3 provides a summary of the ebnergy consumption in joules accounting for the FedHealthFog framework compared with benchmark algorithms. It can be observed that for the training set, the proposed FedHealthFog model subtended an average percentage decrease in the energy consumption over varying number of clients with values 58.48%, 42.44%, and 17.89% as compared to the benchmark models i.e., Hierarchical Fog-Cloud FL, Federated Averaging, and Fed-SGD respectively. Also, the FedHealthFog model showed decrease in energy consumption for the test set as 57.98%, 34.36%, and 35.37% respectively, for the Hierarchical Fog-Cloud FL, Federated Averaging, and Fed-SGD models. The minimal energy consumption in joule was accounted for the proposed FedHealthFog framework which outperformed all other benchmark schemes.

**Table 3:** Summary of energy consumption in joules for training set and test set over varying number of clients corresponding to different models.

Models used	No. of Clients (for Training Set)			
	5	10	15	20
Hierarchical Fog-Cloud FL	2.7303	2.7411	2.7489	2.7567
**Proposed FedHealthFog**	1.0104	1.1326	1.1940	**1.2200**
Federated Averaging	1.7688	1.9187	2.1023	2.1269
Fed-SGD	1.1990	1.3824	1.4787	1.4899
	**No. of Clients (for Test Set)**			
	**5**	**10**	**15**	**20**
Hierarchical Fog-Cloud FL	1.8212	1.8341	1.8409	1.8537
**Proposed FedHealthFog**	0.7591	0.7682	0.7714	**0.7897**
Federated Averaging	1.1546	1.1729	1.1833	1.1942
Fed-SGD	1.1649	1.1899	1.2041	1.2199

## 7 Conclusions and future Scope

We exclusively designed and developed the FedHealthFog framework for resource-constrained edge devices as part of this research project. This framework's deliberate lack of any fixed central institution labeled as a global aggregator is one of its distinguishing characteristics. Instead, we've created strategically placed geospatially located fog nodes to play the job of gathering local updates and then orchestrating global aggregations within this intermediary layer. This creative application of fog nodes has the extra benefit of making it easier to separate and share models depending on the demographic traits of various geographical locations. Moreover, we developed a novel greedy heuristic strategy to improve the system's efficiency. This strategy aims to dynamically choose the best fog node to act as the global aggregator during each operational round.

Our research projects have been supported by a wide range of experimental and simulation studies. These findings culminated in a thorough comparative analysis where we compared FedHealthFog's performance to those of cutting-edge solutions in the industry. The conclusions drawn from our thorough evaluations are significant. FedHealthFog significantly reduces delay, outperforming Hierarchical Fog-Cloud FL, Federated Averaging, and Fog-SGD by 87.01%, 26.90%, and 71.74%, respectively for the test set. Also, the energy consumption for the test set witnessed a reduction of 57.98%, 34.36%, and 35.37% respectively for the three benchmark algorithms analyzed in this study. This notable increase in energy efficiency highlights the FedHealthFog framework's applicability and sustainability. Furthermore, different client fractions and local aggregate data are included in our comprehensive investigation. It turns out that performance is improved when the local aggregate number is less than or equal to 10. These results contribute to the framework's robustness and adaptability by offering important new insights into how it functions in various settings.

The proposed FedHealthFog architecture presents challenges related to uncertain and ambiguous information, which in turn offer potential for enhancing data quality management, risk monitoring, decision-making analytics [[38], [39], [40]], transparency, and continuous learning [41,42]. Future research should focus on utilizing these opportunities to enhance the framework for effectively addressing uncertainty in the healthcare industry [43]. Additionally, it is imperative to build a structure for ongoing innovations. Subsequent research might explore methods for the FedHealthFog framework to develop and improve its answers over time, by leveraging insights from ambiguous information and feedback to inform its learning process.

## CRediT authorship contribution statement

**Subhranshu Sekhar Tripathy:** Formal analysis, Data curation, Conceptualization. **Sujit Bebortta:** Methodology, Formal analysis, Data curation, Conceptualization. **Chiranji Lal Chowdhary:** Resources, Methodology, Investigation, Formal analysis, Conceptualization. **Tanmay Mukherjee:** Investigation, Formal analysis, Data curation, Conceptualization. **SeongKi Kim:** Validation, Supervision, Resources, Conceptualization. **Jana Shafi:** Visualization, Validation, Software, Data curation, Conceptualization. **Muhammad Fazal Ijaz:** Writing – review & editing, Visualization, Validation, Supervision, Resources, Project administration.

## Declaration of competing interest

The authors declare that they have no known competing financial interests or personal relationships that could have appeared to influence the work reported in this paper.
